# Health Advertising on Short-Video Social Media: A Study on User Attitudes Based on the Extended Technology Acceptance Model

**DOI:** 10.3390/ijerph17051501

**Published:** 2020-02-26

**Authors:** Jie Zhao, Jianfei Wang

**Affiliations:** 1 School of Business, Anhui University, Hefei 230601, China; m17201037@stu.ahu.edu.cn; 2 School of Management, Hefei University of Technology, Hefei 230602, China

**Keywords:** public health, short video, social network, social media, TAM

## Abstract

The rapid development of short-video social network platforms provides us with an opportunity to conduct health-related advertising and recommendation. However, so far, there are no empirical evidence on whether users are willing to accept health-related short-video advertisements. Here, acceptance refers to purchase intention, meaning that users will read short-video ads, share ads with others, or even open the product link embedded in ads to purchase the product. In this paper, we make the first attempt to model and quantify user acceptance of health-related short-video advertisements. Particularly, we propose a new research model that enhances the Technology Acceptance Model (TAM) with two new designs. First, we propose four new antecedents including social interaction, intrusiveness, informativeness, and relevance into the original TAM to reflect the features of short-video social networks. Second, we introduce two mediator variables including perceived usefulness and attitude so that we can better study how different factors affect user acceptance of health-related short-video ads. We perform a survey on the Internet and conduct an empirical analysis of the surveyed data. The results show that the four antecedents as well as the perceived ease of use have significant influences on perceived usefulness, attitude, and purchase intention. Further, perceived usefulness plays a valid mediating role in attitude and purchase intention. We also found that users’ perceived ease of use on health-related short-video ads cannot significantly predict users’ attitudes toward ads. This is a new finding in social media-oriented ads. Finally, we integrate the empirical findings and present reasonable suggestions for advertisers and marketers to promote health-related short-video ads.

## 1. Introduction

**Figure 1:**
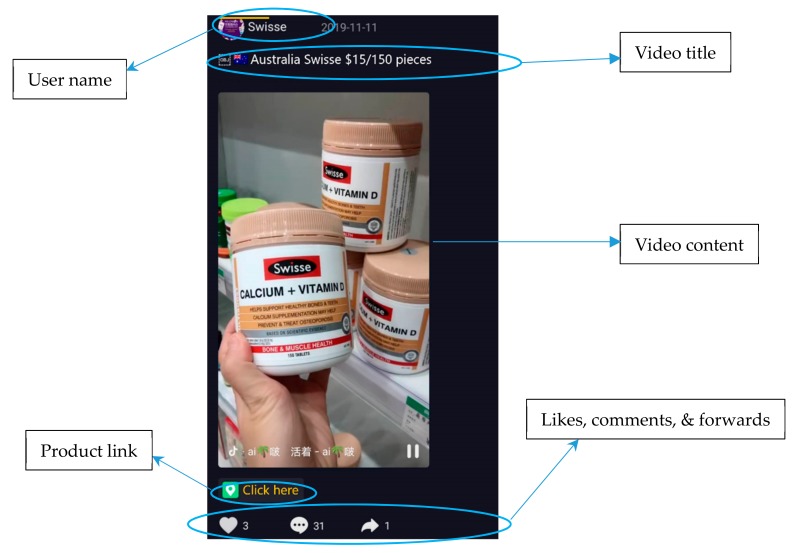
An example of health-related short videos on Douyin (http://www.douyin.com).

Social media is one of the most promising tools in the digital advertising environment [1,2]. Since 2016, short video has become an important field for the expansion of social media such as Tik Tok, Facebook live, YouTube, Snapchat, Instagram, Douyin (Chinese), and Douyu (Chinese). These real-time interactive information platforms provide a free and easy-to-access online resource for live videos for anchors and viewers. For example, Figure 1 shows a typical short video (15 s by default) on the largest Chinese live-video platform Douyin (https://www.douyin.com/), where people can use self-generated videos to share with others something interesting, such as new products and nice places. In Figure 1, the user is introducing a new Swisse product from Australia. So far, short video has gradually developed into a popular social media channel. For example, a recent report showed that the number of short-video users in China had reached 422 million [3].

The rapid development of short-video platforms introduces new opportunities for health promotion, e.g., health-related advertising and recommendation. Compared with traditional social media, short-video platforms have little time delay and can support one-on-one as well as many-to-many social interaction by videos and the barrage (scrolling texts on the screen). For example, the largest Chinese live-video platform Douyin (https://www.douyin.com/) provides one-on-one video interaction that allows one user discusses some topics with another user through live video. At the same time, other users can watch the live video and interact with others via barrages, text reviews, emojis, and images. This new form of social interaction on short-video social network platforms has been demonstrated to be an effective way of advertising [4]. However, in health-related short-video platforms, an important issue is to know users’ intentions of accepting health-related short-video advertisements.

Therefore, this study attempts to investigate whether and how short-video ads affect users’ purchase intentions on health-related products. Particularly, we take the Technology Acceptance Model (TAM) as the base model and extend social interaction, intrusiveness, informativeness, and relevance as four new antecedents to the perceived usefulness of health-related short-video ads. Then, we set perceived usefulness and attitude as two mediate factors and validate their mediating effect in the model. Finally, we conduct an empirical analysis of questionnaire data from the Internet and short-video social network platforms based on the structural equation model. Briefly, following the research framework of the TAM model, we aim to study the following research questions:

RQ1: What are the key factors that affect the perceived usefulness of health-related short-video ads?
RQ2: How do the factors affect the perceived usefulness of health-related ads?
RQ3: How do perceived usefulness and perceived ease of use affect user attitudes and purchase intention on health-related short-video ads?
RQ4: What kind of relationship exists between user attitudes toward health-related short-video ads and users’ purchase intention on health-related products?
RQ5:What is the mediating effect of perceived usefulness and user attitudes to users’ purchase intention on health-related products?

In summary, we make the following contributions in this paper:

(1) We extend the Technology Acceptance Model (TAM) with new antecedents including social interaction, intrusiveness, informativeness, and relevance to analyze the users’ response to health-related short-video ads. To the best of our knowledge, this is the first study that extends the TAM model to analyze user acceptance of health-related short-video ads.
(2) We conduct a survey on the Internet and short-video social network platforms and perform systematical data analysis over the surveyed data. The data analysis consists of many aspects, including measurement model evaluation, structural model evaluation, and mediating effect analysis. The results show that social interaction, intrusiveness, informativeness, relevance, and the TAM factors (perceived usefulness and perceived ease of use) have a significant influence on user acceptance of health-related short-video ads. Further, we find that users’ perceived ease of use on health-related short-video ads cannot significantly predict users’ attitudes toward ads. This is a new finding that is contrary to previous studies in social media-oriented ads.
(3) We integrate the empirical findings and present reasonable suggestions for advertisers and marketers to better develop health-related short-video ads.

The remainder of this paper is structured as follows. Section 2 describes the related work and the differences between previous studies and this paper. Section 3 presents the research model as well as the hypothesis propositions. Section 4 describes the data collection and measurements. Section 5 presents the results of data analysis. Section 6 discusses the research implications and suggestions. Finally, in Section 7, we conclude the entire paper.

## 2. Related Work

### 2.1. Short-Video Advertising

Digital advertising significantly influences users’ attitudes and intentions to purchase [5]. There are many studies about the factors that influence the effectiveness of online advertising [6], video advertising [7], and social media advertising [2]. Although short-video ads are a promising form of online advertising, this ad form has not been adequately examined in the research. The core of short-video ads is “video + sociality”, and it has become an important field for the expansion of social media. Therefore, we study our questions mainly based on the research of social media advertising. Some quote video studies, of course.

There have been some existing studies on informativeness and intrusiveness and how such concerns may influence users’ attitudes toward advertising and product purchase intentions [8,9,10,11,12,13,14,15,16]. Generally, informativeness is defined as the information value provided by media like texts, images, or videos [11] and intrusiveness is defined as the degree to which people deem the presentation of information as contrary to their goals [14]. Both informativeness and intrusiveness have been regarded as key factors to predict the helpfulness of messages in the research of social networks. For example, Dehghani et al. [12] conducted an empirical study on YouTube ads and found that a high value of informativeness led to a high level of user acceptance of YouTube ads, meaning that informativeness is a positive factor affecting users’ attitude toward YouTube ads. Meanwhile, they found intrusiveness is a negative factor, meaning that a high value of intrusiveness led to a low level of user acceptance of YouTube ads. Lee and Hong empirically investigated that informativeness has a potential contribution to a positive online behavior based on the theory of reasoned action and the social influence theory [13]. Jung found perceived ad relevance influences advertising effectiveness while it could increase privacy concerns, which ultimately raise ad avoidance in social media [16].

Relevance is another factor that may affect user acceptance of advertising. Relevance has been widely adopted in web search and online recommendation [17]. For instance, a web search engine can rank the searching results according to the relevance between the user query and the returned web pages. In the online recommendation in E-commerce, the relevance between the user profile and products is usually used to predict the items that may fit within the users’ interests. Similarly, the relevance between a user’s interest and short-video advertisements may also affect the user acceptance of short-video ads [18]. Therefore, we will also consider relevance as a construct in the research model of this study, which will be detailed in Section 3.

On the other hand, according to the social influence theory [19], a high level of social interactions can enhance the feelings of affection, trust, belongingness, and warmth. Currently, most people are engaged in short videos by mobile apps. The mobile short-video apps offer people real-time interaction with other ones. In addition, platforms like Douyin and Tik Tok allow users to interact with short videos. For example, users can click the product link and fill a form to deliver their purchase interest to vendors. Therefore, social interaction can be a key factor affecting user acceptance of short-video ads.

So far, short-video ads are still in the early developing stage. Existing work has paid little attention to how the new features of short videos impact users’ attitudes toward purchase behavior. Based on previous experiences in social media ads, many researchers claimed that two aspects of information should be considered in the context of social media ads [20,21,22]. The first one, product information, focuses on informativeness, relevance, and intrusiveness of information [20]. The second one, personal interaction, refers to social interaction, which reflects the impact of social identity and group norms on community users’ group intention of accepting advertising [21]. Windels et al. examined the differences between native advertisements and friend referrals on social networking sites. They found that social relationships did not always work [22]. In this paper, we consider these related studies as well as the features of short-video ads to design antecedents toward the perceived usefulness of users and the attitude to short-video ads. As a result, four antecedents including informativeness, relevance, intrusiveness, and social interaction are designed in our study.

### 2.2. Technology Adoption Model (TAM)

The Technology Acceptance Model (TAM) was developed to describe users’ behavior to accept or reject the use of new technologies [23]. The TAM model defines two variables, namely perceived usefulness and perceived ease of use, to quantify user attitude to information technology, which in turn can be used to measure user acceptance of information technologies.

Although the TAM model is initially designed to explain and predict the behavior of individuals on the use of information systems, it has been used in many studies [24,25,26]. Previous studies have demonstrated the applicability of the TAM model to online advertising. For example, Demangeot and Broderick examined a modified technology acceptance model (TAM) that was developed to test the intention to use SMS advertising [27]. Based on the ability of TAM and short video including a wide variety of online media services and SNS, it is a reasonable choice to consider the TAM model to model and quantify user acceptance of short-video ads.

The TAM model has been widely adopted in many existing studies. In this paper, we also adopt the TAM model as the basic research model. There are two reasons. First, many studies in social media have applied the TAM model to analyze the acceptance factors of online advertising and social network advertising. The research scope of this paper, i.e., short video, has the characteristics of both online media and social networks; thus, it is a reasonable choice to select the TAM model. Second, the basic factors in the TAM model, namely perceived usefulness (PU) and perceptual ease of use (PEOU), are suitable to distinguish the usefulness of live advertising and the impact of live-video technologies.

On the other hand, differing from the traditional TAM model, we integrate social interaction, intrusiveness, informativeness, and relevance with TAM and propose to use perceived usefulness as the mediate factor, forming an augmented TAM model that is more suitable for studying the intact path of short-video ads. Even though research integrating these external variables with the TAM is limited, available empirical findings generally support the influence of these factors on perceptions about users’ ad attributes and adoption intentions.

In addition to the TAM model, another widely used model for predicting user acceptance of short-video ads is the Unified Theory of Acceptance and Use of Technology (UTAUT) model [28], which was proposed to incorporate various models of human behavior theory. The UTAUT model was constructed by extracting three variables that affect users’ behavioral intentions, one variable that influences action, and four moderators that mediate the effects of the process. Some of the variables had similar concepts with variables that construct the TAM model. One major difference between UTAUT and TAM was that UTAUT proposed four control variables (i.e., gender, age, experience, and voluntariness) to further enhance the predictive power of the model. Based on UTAUT, Venkatesh et al. incorporated three other constructs into UTAUT, namely hedonic motivation, price value, and habit, extending UTAUT into UTAUT 2 [29]. Compared to UTAUT, the extensions proposed in UTAUT2 produced a substantial improvement in the variance explained in behavioral intention. As a result, both UTAUT and UTAUT 2 have been actively used in predicting users’ purchase behavior in many areas like healthcare systems [30] and social-network-based advertising [1]. However, as suggested by Venkatesh et al. [29], in order to increase the applicability of the original UTAUT as well as the UTAUT 2 model, other relevant factors are usually needed to be extended to meet the specific requirements of different applications, technologies, countries, etc.

In summary, both TAM and UTAUT are actively utilized in the research of user adoption of new technologies. Whether or not to use TAM or UTAUT mainly depends on the particular type of the application. In this paper, we select TAM rather than UTAUT as the basic model for analyzing user acceptance of health-related short-video ads for the following reasons. First, UTAUT is a behavioral model aiming to explain the behavior of people in the use of information systems [31], but in this study, the content of short-videos, as measured by the antecedents named informativeness, relevance, and intrusiveness in our model, is also an important factor. Second, the UTAUT model is more complex than TAM; thus, extending TAM is easier than extending UTAUT. Nevertheless, as Venkatesh et al. [29] and Dwivedi et al. [32] reported, most studies only employed a subset of the UTAUT model, and the moderators were typically removed. To this end, the TAM model can have similar predictive power as the UTAUT model without moderators. However, it is still worth investigating the extension of the UTAUT or UTAUT 2 model to predict the user adoption of health-related short-video ads in the future.

## 3. Research Model and Hypotheses

### 3.1. Research Model

**Figure 2:**
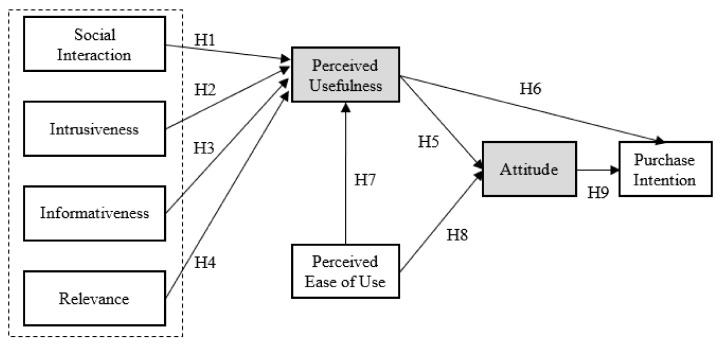
The research model extended from Technology Acceptance Model (TAM) [23] (each rectangle represents one variable. The mediator variables are shaded grey. Purchase intention is the dependent variable, and the remaining five are independent variables).

In this paper, we first selected the main factors in the TAM model as basic independent variables, i.e., perceived usefulness (PU), perceived ease of use (PE), and ad attitudes (AT). Further, we introduced social interaction (SI), informativeness (IR), intrusiveness (IN), and relevance (RE) as new antecedents of perceived usefulness. The selection of the four factors (i.e., SI, IR, IN, RE) was mostly based on two aspects. First, previous studies in social media have shown that these factors are critical to the usefulness of social media advertising [2,12,16]. Second, these factors are suitable for short-video ads, as they reflect the information features of live video ads. Thus, it is reasonable to introduce them to the research model. As a result, the perceived usefulness (PU) in our research model became a mediator variable. The purchase intention (PI) was set to be the dependent variable. In the study of behavior intention, attitude has been commonly recognized as a variable that affects the willingness of behavior. Thus, we chose attitude as another mediator variable. On this basis, this paper put forward the research model of user responses to health-related short-video advertisements. Figure 2 shows the components of the proposed research model.

### 3.2. Research Hypothesis

According to the research model, there are three independent variables, namely perceived usefulness (PU), perceived ease of use (PE), and ad attitude (AT), and four antecedent variables, namely social interaction (SI), informativeness (IR), intrusiveness (IN), and relevance (RE). In addition, there are two mediator variables called perceived usefulness (PU) and ad attitude (AT). These variables are supposed to impact the dependent variable named purchase intention (PI).

To reveal the relationships among these factors, we propose hypotheses to the research questions presented earlier, as listed in Table 1. The right column in Table 1 shows the corresponding hypotheses that are proposed to answer the research question. In the following subsections, we will explain each hypothesis.

Sociability has been defined as ‘the extent to which the computer-mediated communication environment facilitates the emergence of social space by allowing social affordance [33]. Social interaction can mitigate users’ perceptions of ad avoidance and enhance the effectiveness of ads [19]. Interactive online media are an increasingly preferred format for users and advertisers. Particularly, health-related short-video ads are more social than traditional social media advertising, whereby audiences can, in a timely manner, view, share, and post barrages on these ads with anchors and peer audiences. Given the massively real-time interactivity distinguishing live video from traditional social media, real-time interactivity may enhance the influence of social interactions or depress viewers’ experience because of barrages filled with mobile screens. Therefore, we make the following hypothesis:

**Hypothesis** **1** **(H1)**. *Social interaction of health-related short-video ads positively affects users’ perceived usefulness (PU) of ads.*

Intrusiveness describes the extent to which the content is messy and irritating to users [34]. Intrusiveness in social media advertising will be negatively related to the perceived usefulness of ads [2,14,15]. When an inconsistent ad is posted with live videos, audiences may spend efforts on non-major tasks and cannot effectively deal with advertising information, resulting in the ineffectiveness of advertising. In addition, the small screen of a mobile phone may also make intrusiveness be a negative factor in live video ads. Moreover, intrusive ads may bring the negative emotion to users [35]. Hence, we make Hypothesis 2:

**Hypothesis** **2a** **(H2a)**. *The intrusiveness of health-related short-video ads negatively affects users’ perceived usefulness (PU) of ads.*

**Hypothesis** **2b** **(H2b)**. *The intrusiveness of health-related short-video ads negatively affects users’ perceived ease of use of ads.*

Informativeness represents the information that is helpful and resourceful [36]. It can inform users about product alternatives. Consumers tend to gain information more through unconditional, interpersonal information exchange [37]. Studies have found informativeness to be important in the formation of consumer attitudes to electronic commerce websites as well as to SNS advertising [10] and to social media advertising [13]. The informative content of a message positively influences the perceived value of online advertising [15]. Hence, we make Hypothesis 3:

**Hypothesis** **3** **(H3)**. *Informativeness of health-related short-video ads positively affects users’ perceived usefulness (PU) of ads.*

Relevance is used to capture users’ general perception of similarity between short-video ads and live video content. According to the limited capacity model of attention [18], the total capacity allocated to process activities can be divided into the primary capacity for the most important task and the spare capacity for less important tasks. When an inconsistent ad is placed in the primary task field, audiences may reduce the likelihood of primary capability being allocated to processing the ad. Advertising relevance decreases ad skepticism and ad avoidance in e-mails, direct mails, telemarketing, and text messages [38]. Hence, we make Hypothesis 4:

**Hypothesis** **4** **(H4)**. *The relevance of health-related short-video ads positively affects users’ perceived usefulness (PU) of ads.*

Perceived usefulness (PU) refers to the degree to which individuals perceive the use of health-related short-video ads. If users think that short-video ads are useful, they are much likely to have a positive attitude on using them. Attracting and motivating short-video ads can also lead to purchase intention [2]. Hence, we make Hypotheses 5 and 6 as follows:

**Hypothesis** **5** **(H5)**. *Users’ perceived usefulness of health-related short-video ads positively affects their attitudes toward ads.*

**Hypothesis** **6** **(H6)**. *Users’ perceived usefulness of health-related short-video ads positively affects their purchase intentions*.

Perceived ease of use (PEOU) is defined as the mobile phone users’ expectations about the effort required to use live video advertising messages. Short-video advertising requires some cognitive effort from users such as maneuvering through screens, reading the advertising, and making a quick evaluation of whether it is worthy (in terms of time or effort) to take further action. Therefore, the easier they perceive using short-video advertisements to be in general, the more likely they are to later engage in the action implied by the ad. Hence, we make Hypotheses 7 and 8:

**Hypothesis** **7** **(H7)**. *Users’ perceived ease of use of health-related short-video ads positively affects their perceived usefulness.*

**Hypothesis** **8** **(H8)**. *Users’ perceived ease of use of health-related short-video ads positively affects their attitudes toward ads.*

Attitude towards advertising is regarded as an important factor for advertising management because consumers’ attitude can influence purchase intention [39]. Previous research has indicated that purchase intention is a critical indicator of advertising effectiveness and may be affected by indicators such as attitude towards ads [2,12,36]. For example, Lin and Kim found that the attitude towards ads affected brand awareness and purchase intentions [2]. Tran found that purchase intention was positively affected by ad attitude on Facebook [40]. Whether consumers prefer a product or an advertisement depends on the informativeness of the advertisement, i.e., whether the advertisement can provide the opportunity and convenient ways for purchase. Therefore, the informational nature of advertisement affects the consumer’s purchase intention. Based on these observations, we make the following hypothesis:

**Hypothesis** **9** **(H9)**. *Users’ attitudes of health-related short-video ads positively affect their purchase intentions.*

Note that we only set up the Hypotheses H1–H4 to measure the impact of the four constructs on PU but not on PEOU. This is mainly because the constructs are all about the information offered by advertisements, but not about the operations or behavior of advertising. On the other hand, the perceived ease of use (PEOU) refers to the ease of operations that users perceive, i.e., PEOU is operation related. Thus, in this paper, we only focus on the impact of the constructs on PU.

## 4. Data Collection and Measurement

### 4.1. Data Collection

We mainly conducted questionnaires on users who have online shopping experiences due to mobile live ads, so that we could gain useful feedback from experienced users on short-video platforms. The data for this study were collected using an online survey in Douyu.com (a famous short-video platform in China) during January 2018, which lasted for three weeks. Three hundred and fifty questionnaires were sent to the viewers with anchors’ help, and 289 questionnaires were received. Thirty-three questionnaires that contained inconsistent answers or incomplete information filling were removed from the data set.

Note that it is not possible to ask the Douyu platform to only present health-related short-video ads to the participants, because the platform does not offer the function of filtering health-related short-videos (actually, this could be a complicated task which needs effective algorithms). We offered the participants three weeks to return questionnaires, and all questions were restricted to health-related short-video ads (an example is given in Figure 1). Although we do not know what health-related videos the participants watched, we believe that the involved short-videos covers a wide range of health-related products. This is because the number of short videos on the Douyu platform is dramatically increasing day by day. In addition, different users have various interests, so the platform will recommend different short-video ads to each user.

Specifically, we asked three Douyu anchors for help, namely Prisoner, Valila, and Liu shaji, to distribute the questionnaires to their fans. The time span of the questionnaires was from January 3, 2018 to January 24, 2018. Consequently, we collected 256 valid questionnaires, which met the analytical requirements of the structural equation model.

In the data set, the proportions of males and females were 53.5% and 46.5%, respectively. There were 79.8% users that were under 30 years old. The duration of using live videos ranged from 0 to 2 years, and 58.6% of the users had experienced live videos for 1 to 2 years. Furthermore. 50.4% of the users were undergraduates and graduates (see Table 2). The attributes of the surveyed data were similar to those in the IResearch report (IResearch, The research report on the China mobile live video market, http://wreport.iresearch.cn/uploadfiles/reports/636161408839324949.pdf).

### 4.2. Measurement

The survey was designed using a multi-item approach. All variables were carried out by a five-point Likert-scale, ranging from strongly disagree (1) to strongly agree (5). Items were borrowed from previous literature and modified for the context of this study.

The questionnaire consisted of three parts:

(1) Sample Selection: Users who have online shopping experiences due to mobile live ads are considered as samples in this study.
(2) Sample Characteristics: This part mainly measures the sample statistical data of participants.
(3) Variable Questionnaire: This study included eight latent variables, including Social interaction (SI), Intrusiveness (IR), Informativeness (IN), Relevance (RE), Perceived Usefulness (PU), Perceived ease of use (PE), Ad attitudes (AT), and Purchase Intention (PI).

The whole items are shown in Table 3.

## 5. Data Analysis and Results

The research model was tested using AMOS 21, a structural modeling technique that is well suited for estimating parameters and theoretical models. In Section 5.1, we use confirmatory factor analysis (CFA) to validate the reliability and validity of the measurement model. In Section 5.2, we perform maximum likelihood estimation (MLE) and the bootstrapping method to examine the structural model. In Section 5.3, we measure the mediating effects of PU and AT in the research model. In Section 5.4, we summarize the conclusion of the research.

Before measuring the model, we examined whether common method bias was a concern in this study with two tests of common method variance (CMV) [44]. First, an exploratory factor analysis of all items extracted eight factors, which explained 73.72% of all the variance, with no single factor accounting for significant loadings (at the *p* < 0.10 level) for all items. Second, single method-factor approaches indicated that there was no significant difference among the method factors (ΔCMIN/DF = −0.032, ΔGFI = 0.009, ΔAGFI = 0.001, ΔIFI = 0.003, ΔRMSEA = −0.002). Thus, we concluded that CMV was not a concern in this data set.

### 5.1. Measurement Model Evaluation

The measurement model evaluation mainly included three parts, namely reliability analysis, convergent validity, and discriminant validity.

Reliability is usually examined by using internal consistency reliability and composite reliability. As shown in Table 4, the coefficient alpha for the eight variables scored in the range from 0.800 to 0.907 (more than 0.70 [45]). The composite reliability (CR) of all constructs was above 0.70 [46]. The reliability of the model was achieved.

Convergent validity is usually examined by using composite reliability (CR) and average variance extracted (AVE). As shown in Table 4, all items load significantly on their respective constructs and none of the loadings is below the cutoff value of 0.70. AVE of each variable was more than 0.50 [47]. Thus, convergent validity was achieved.

Discriminant validity is analyzed to examine whether a measurement is not a reflection of any other measurement or not. As shown in Table 5, the square root of AVE for each variable is greater than the other correlation coefficients. The discriminant validity of the model is achieved.

### 5.2. Structural Model Evaluation

The research model was tested by using AMOS 23, and a bootstrapping resampling procedure (2000 samples) was used to ensure the solidity.

The resulting indices indicated a good model fit (x2/df = 1.318; GFI = 0.903; RMSEA = 0.035; NFI = 0.909; CFI = 0.976; TLI = 0.972; RMR = 0.071; SRMR = 0.058).

**Figure 3:**
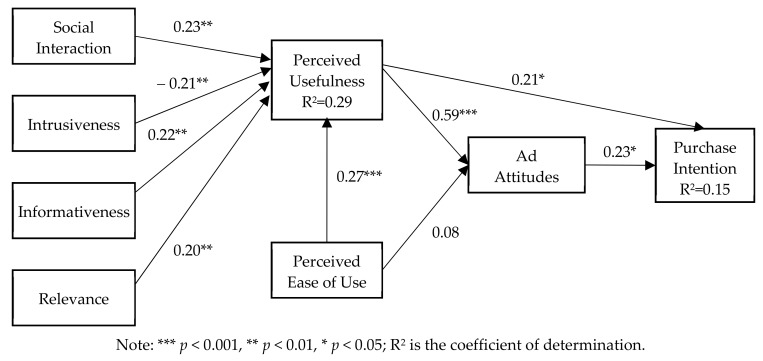
Results of structural model evaluation.

Figure 3 shows that social interaction (SI), informativeness (IN), and relevance (RE) of health-related short-video ads were significant and positive predictors of perceive usefulness of ads (β = 0.23, *p* < 0.01; β = 0.22, *p* < 0.01; β = 0.20, *p* < 0.01). These findings support H1, H3, and H4, respectively, and this supports previous studies [12,19]. Similarly, intrusiveness (IR) of health-related short-video ads was a significant and negative predictor of the perceive usefulness of ads (β = −0.21, *p* < 0.01), and H2 is validated. This is consistent with previous research [2,15]. These four factors (SI, IR, IN, and RE) explained 29% of the variance in the perceived usefulness of health-related short-video ads. Additionally, comparing these four coefficients, we found social interaction was more important to users’ perceived usefulness of ads. Users’ perceived usefulness of health-related short-video ads positively affected their attitudes toward ads (β = 0.59, *p* < 0.001) and purchase intention (β = 0.21, *p* < 0.05); these results validate H5 and H6. Users’ perceived ease of use of health-related short-video ads was a significant and positive predictor of perceived usefulness of ads (β = 0.27, *p* < 0.001), and H7 is validated. However, users’ perceived ease of use of health-related short-video ads could not significantly predict users’ attitudes toward ads. H8 is not validated and this is not consistent with Lin and Kim’ research, as they found users’ perceived ease of use of social media ads significantly predict users’ attitudes [2]. This enlightens us that perceived ease of use may not be an important factor in health-related short-video ads. Therefore, only users’ perceived usefulness of ads explained 37% of the variance in attitudes toward ads. Finally, users’ attitudes of health-related short-video ads were found to positively predict their purchase intentions (β = 0.23, *p* < 0.05), and H9 is validated. Users’ perceived usefulness of ads and attitudes toward ads accounted for 33% of the variance in purchase intentions.

### 5.3. Mediating Effects Analysis

Mediation analyses were tested using the Preacher and Hayes’ bootstrapping (2000 bootstrap samples) analysis with the PROCESS macro [48]. We focused on two mediate variables (perceived usefulness and ad attitudes) to find the impact path of user responses to health-related short-video advertisements because users’ perceived ease of use of health-related short-video ads cannot significantly predict users’ attitudes toward ads. The results are shown in Table 6.

The mediating path (Social Interaction→PU→AT) tested the specific indirect effect of social interaction of ads on ad attitudes through perceived usefulness of ads. As both the upper and lower limits of the confidence interval (CI) were above zero (0.014, 0.166), this indirect effect can be interpreted as significantly positive. Similarly, the direct effect of the perceived usefulness of ads can be interpreted as significantly positive. Hence, the perceived usefulness of ads served as a partial mediating variable between social interaction and ad attitudes. That means those who perceived the sociality of health-related short-video ads believed that the ad was more useful, and this increased usefulness enhances the viewers’ positive attitudes toward the ad.

The mediating path (Intrusiveness→PU→AT) tested the specific indirect effect of intrusiveness of ads on ad attitudes through perceived usefulness of ads. As both the upper and lower limits of the confidence interval (CI) were above zero (−0.134, −0.018), this indirect effect can be interpreted as significantly positive. The perceived usefulness of ads served as a partial mediating variable between intrusiveness and ad attitudes. That means those who perceived intrusiveness of health-related short-video ads believed that the ad was less useful, and this decreased usefulness enhances the viewers’ negative attitudes toward the ad.

The mediating path (Informativeness→PU→AT) tested the specific indirect effect of informativeness of ads on ad attitudes through perceived usefulness of ads. Perceived usefulness of ads served as a partial mediating variable between informativeness and ad attitudes (CI: 0.011, 0.164). That means those who perceived informativeness of health-related short-video ads believed that the ad was more useful, and this increased usefulness enhances the viewers’ positive attitudes toward the ad.

The mediating path (PEOU→PU→AT) tested the specific indirect effect of perceived ease of use of ads on ad attitudes through perceived usefulness of ads. Perceived usefulness of ads served as a partial mediating variable between perceived ease of use and ad attitudes (CI: 0.024, 0.188). That means those who perceived ease of use of health-related short-video ads believed that the ad was more useful, and this increased usefulness enhances the viewers’ positive attitudes toward the ad.

Similarly, the perceived usefulness of ads served as a partial mediating variable between perceived ease of use and purchase intentions (CI: 0.018, 0.195). That means those who perceived ease of use of health-related short-video ads believed that the ad was more useful, and this increased usefulness enhances the viewers’ purchase intentions.

Finally, the mediating path (PU→AT→PI) tested the specific indirect effect of perceived usefulness of ads on purchase intention through ad attitudes. The indirect effect was significant (CI: 0.026, 0.368) while the direct effect was not significant (CI: −0.007, 0.511). Hence, ad attitudes served as a full mediating variable between perceived usefulness and purchase intention. That means users’ attitude toward health-related short-video ads was a key path to the effectiveness of ads.

In summary, there were four impact paths of user responses to health-related short-video advertisements. First, social interaction had an indirect effect on purchase intentions through perceived usefulness and ad attitudes in serial (SI→PU→AT→PI). Second, intrusiveness had an indirect effect on purchase intentions through perceived usefulness and ad attitudes in serial (IR→PU→AT→PI). Third, informativeness had an indirect effect on purchase intentions through perceived usefulness and ad attitudes in serial (IN→PU→AT→PI). Fourth, perceived ease of use had an indirect effect on purchase intentions through perceived usefulness and ad attitudes in serial (PE→PU→AT→PI).

### 5.4. Summary of Hypothesis Validation

By combing the results described in Section 5.1–5.3, we present the summary of the hypothesis validation in Table 7.

The validation-result column in Table 7 shows the final validation results of each hypothesis, from which we can see that H1-H4 are well established. These hypotheses correspond with the research questions RQ1 and RQ2 presented in Table 1. This implies that five qualities (social interaction, intrusiveness, informativeness, relevance, and perceived ease of use) of health-related short-video ads help enhance the perceived usefulness of ads. Among all the factors, according to different coefficients, social interaction of health-related short-video ads made a more valuable contribution to advertising usefulness. Thus, advertisers and marketers should not only focus on social interaction, intrusiveness, informativeness, relevance, and the perceived ease of use of health-related short-video ads, but also pay more attention to social interaction.

Regarding hypotheses H5, H7, and H8, which aim to answer RQ3, only the perceived usefulness of health-related short-video ads helped produce positive user attitudes. H9 answers RQ4, finding that positive user attitudes significantly promotef purchase intentions. Combined with the analysis of the mediating effects in Section 5.3, we found four impact paths of user responses to health-related short-video advertisements (SI→PU→AT→PI; IR→PU→AT→PI; IN→PU→AT→PI; PE→PU→AT→PI). This answers RQ5. Thus, advertisers and marketers may maximize resource utilization through the impact paths.

## 6. Discussions

### 6.1. Research Implications

(1) The study of this paper is based on the background of online ads in the health-related short video, which has become an important field for the expansion of social media and a part of people’s daily life. Due to the value of short-video ads, it is necessary to explore user responses to short-video advertisements. We propose a research model based on the integration of the TAM model, social interaction, intrusiveness, informativeness, and relevance. This model augments the application of the widely used TAM model and offers referential values for other related researches. In addition, we present empirical results on user acceptance of participating in health-related short-video ads. These results can provide new research insights for advancing health-related short-video advertisements, e.g., a socially interactive mechanism for mobile ads.
(2) This paper studies users’ purchase intention towards health-related short-video ads and is valuable for advertisers and marketers to realize the importance of developing health-related short-video advertisements. With the rapid development of technologies and social entertainment, users’ attitudes toward online ads may have changed a lot. Short video has been a promising tool for seeking business opportunities and establishing brand expression, and advertisers need to keep reforming their advertising strategy to meet user needs.
(3) According to the empirical study conducted in this paper, the five factors (social interaction, intrusiveness, informativeness, relevance, and perceived ease of use) defined in the research model are helpful to enhance the perceived usefulness of health-related short-video ads, which can in turn affect user acceptance of health-related short-video ads. The results indicated that social interaction, informativeness, and relevance of advertisements are all positive factors, while intrusiveness is a negative factor, meaning that the increasing of intrusiveness will lower the user acceptance of health-related short-video ads. These results are consistent to what were revealed by previous studies [12,15,19]. Furthermore, compared with other factors, social interaction showed a higher impact to the perceived usefulness of short-video ads. This is a new finding of the study. While advertisers should be mindful of multiple aspects of ads, including intrusiveness, informativeness, relevance, and perceived ease of use, they should largely focus their efforts on the social interactions that stem from these ads.
(4) We also find that users’ perceived ease of use of health-related short-video ads cannot significantly predict users’ attitudes toward short-video ads. This does not support Lin and Kim’ research conclusions [2], i.e., users’ perceived ease of use of social media ads significantly predict users’ attitudes toward ads. To this end, the perceived ease of use may not be an important factor in health-related short-video ads.
(5) We find four impact paths of user responses to health-related short-video advertisements (SI→PU→AT→PI; IR→PU→AT→PI; IN→PU→AT→PI; PE→PU→AT→PI) by mediating effects analysis. Thus, advertisers and marketers can maximize resource utilization through the impact paths and promote the effectiveness of health-related short-video ads.

### 6.2. Suggestions

The empirical analysis of this study can offer some useful hints for advertisers to advance short-video ads. Below, we present some suggestions for advertisers and marketers to better develop health-related short-video ads:

(1) Consider various factors when promoting short-video ads.

The results of this study show that the five factors (social interaction, intrusiveness, informativeness, relevance, and perceived ease of use) defined in the model can all positively affect the perceived usefulness of short-video ads. Thus, we suggest that advertisers should consider all five factors rather than focusing on one or some specific factors. For example, if advertisers neglect the ‘relevance’ factor, they might issue short-video ads in which the video content is irrelevant to the topic of the ads.

(2) Emphasize the social interactions of short-video ads.

Our study shows that social interaction is more influential to the perceived usefulness of short-video ads compared with other factors. Thus, we suggest advertisers to pay more attention to the social interactions of short-video ads, which is a shortcoming of current short-video ads. As shown in Figure 1, current short-video ads provide few ways for social interaction. They are much like an introduction of a product, which neglect the use of social interaction. However, the results of this study show that social interaction is a key construct for enhance user acceptance of short-video ads. Therefore, we strongly suggest advertisers to design social-interaction tools in short-video ads. For example, they can embed a voting button in a video by using new technologies like HTML5 to let video viewers submit their choices.

(3) Focus on users’ perceived usefulness of short-video ads.

Through the mediating factor analysis, we found that users’ perceived usefulness of short-video ads plays an important role in the impact path from the influential factors to users’ purchase intention. Therefore, enhancing users’ perceived usefulness of short-video ads is the key point of improving the effectiveness of short-video ads. On the one hand, advertisers should improve the quality of short-video ads, e.g., by increasing the informativeness and relevance of ads, or by providing new kinds of social interactions to attract users’ attention. On the other hand, advertisers should avoid issuing any false advertisements, which are much harmful to consumers’ confidence on products. Once users find that the short-video ads issued by an advertiser are over-claimed or false, the fast spreading of word-of-mouth on social network platforms will do considerable harm to the reputation of the advertiser.

(4) Advance ads on short-video social network platforms.

Short videos provide a free, real-time, and easy-to-access online way for enterprises to deliver ads to potential users. Recently, the number of the users on short-video social network platforms has shown a dramatical increasing trend, because live videos provide more attractive presentation than conventional textual web pages or tweets. Thus, it is important for enterprises to develop their short-video-based advertising systems and departments to advance ads on short-video social network platforms. Accordingly, the live short video as a new style of advertising could be utilized as a new tool to enhance the market competitiveness of enterprises.

## 7. Conclusions and Future Work

In this paper, we analyze the major factors that influence user responses to health-related short-video advertisements. Particularly, we made three contributions. First, we extended the Technology Acceptance Model (TAM) with new antecedents including social interaction (SI), intrusiveness (IR), informativeness (IN), and relevance (RE) to analyze the users’ response to health-related short-video ads. We also introduced two mediate factors, i.e., perceived usefulness (PU) and ad attitude (AT), to reflect the influence of independent variables on the dependent variable named purchase intention (PI). To the best of our knowledge, this is the first study that extends the TAM model to analyze user acceptance of health-related short-video ads. Second, we conducted a survey on the Internet and short-video social network platforms and performed systematical data analysis over the surveyed data. The data analysis consisted of many aspects, including measurement model evaluation, structural model evaluation, and mediating effect analysis. The results showed that social interaction, intrusiveness, informativeness, relevance, and the TAM factors (perceived usefulness and perceived ease of use) had a significant influence on user acceptance of health-related short-video ads. Further, we found that users’ perceived ease of use on health-related short-video ads could not significantly predict users’ attitudes toward ads. This is a new finding that is contrary to previous studies in social media-oriented ads. Third, we presented reasonable suggestions for advertisers and marketers to better develop health-related short-video ads.

This study is beneficial for advertisers to realize the importance of understanding the effectiveness of health-related short-video ads. Base on the empirical results of this study, we gained some new findings, some of which are contrary to existing research. These findings are helpful to reveal the major factors that influence users’ ad attitudes and intentions to purchase and to quantify the impacts of these factors on them. The suggestions made based on the empirical results of this study can provide some management ideas for advertisers and academia to develop health-related short-video ads.

Some limitations of this study can be summarized as follows. First, although hundreds of valid questionnaires are theoretically enough to conduct data analysis, in this big data era, this number is relatively small to draw reliable and robust conclusions. Second, the results of this study have shown that the four antecedents, as well as the perceived ease of use, have significant influences on perceived usefulness, attitude, and purchase intention towards mobile live ads. However, the inherent theoretical basis has not been revealed yet. Third, the survey in this study has some limitations. Currently, we are only able to survey people on a Chinese short-video platform. The model, as well as the results, may not extend to people in other countries or on other platforms. In addition, as the respondents in the survey mainly cover young people (79.8% of them were under 30 years old), the results may not suit those advertisements that target old people.

Thus, in the future, there are some research issues that are worth further investigating. First, a further study on secondary data collected from some crowdsourcing platforms like Amazon Mechanical Turk [49] could be better to analyze user acceptance of advertising and marketing on short-video platforms. Second, because users’ decision-making behavior could be impacted by other factors, future work can be focused on other possible factors, such as users’ educational background and online experiences. Finally, in addition to the TAM model, it is also worth studying other research models within the big data and online community context [50].

## Figures and Tables

**Table 1:** Research questions and corresponding hypotheses.

Question Number	Research Question	Corresponding Hypotheses
RQ1	What are the key factors of short-video ads?	H1, H2, H3, H4
RQ2	How do the factors affect the perceived usefulness of health-related ads?	H1, H2, H3, H4
RQ3	How do perceived usefulness and perceived ease of use affect user attitudes and purchase intention on health-related short-video ads?	H5, H7, H8
RQ4	What kind of relationship exists between user attitudes toward health-related short-video ads and users’ purchase intention on health-related products?	H9
RQ5	What is the mediating effect of perceived usefulness and user attitudes to users’ purchase intention on health-related products?	H5, H6, H8, H9

**Table 2:** Statistics of the surveyed users.

Measure	Frequency	Percentage (%)
Gender		
female	119	46.5
male	137	53.5
Age		
<30	204	79.8
≥30	52	20.2
Education		
Below bachelor’s	79	30.9
Bachelor’s	129	50.4
Master’s	39	15.2
Ph.D.	9	3.5
Years of using short videos		
Less than 1 year	45	17.6
1–2 years	150	58.6
More than 2 years	61	23.8
Short video Usage		
Everyday	79	30.9
5–6 days per week	70	27.3
3–4 days per week	66	25.8
Once or twice a week	41	16

**Table 3:** Summary of measurement scales constructs (in the table, short video refers to health-related short videos).

Construct	Item	Source
Social Interaction	SI1: Short video ads provide me the opportunity to have lively, interesting, and engaging interaction with others	Animesh et al.(2011) [41]; Fan et al.(2017) [19]
	SI2: In general, I think that short video ads strongly facilitate social interactions	
	SI3: Overall, I am very satisfied with the social aspects of short video ads	
Intrusiveness	IR1: Short video ads are an insult to one’s intelligence	Chowdhury et al. (2010) [42]; Pintado et al. (2017) [15]
	IR2: Short video ads are bothersome	
	IR3: Short video ads are disappointing	
	IR4: Short video ads are irritating	
Informativeness	IN1: Short video ads are a good source of information on products	Chowdhury et al. (2010) [42]; Pintado et al. (2017) [15]
	IN2: Short video ads offer opportune information on products	
	IN3: Short video ads are a good source of updated information	
Relevance	RE1: The presentation style of short video ads is my favorite one	Wang et al. (2013) [43]; Fan et al. (2017) [19]
	RE2: The theme of advertising is very close to my interest	
	RE3: The function of advertising fits well with my needs	
Perceived Usefulness	PU1: Short video ads would enable me to purchase favorite products	Ducoffe (1996) [8]; Lin & Kim (2016) [2]
	PU2: Short video ads would enable me to broaden my understanding of the product or service	
	PU3: Short video ads provide valuable information for products	
Perceived Ease of Use	PE1: Learning how to participate in short video ads would be easy for me (viewing, clicking and commenting)	Ducoffe (1996) [8]; Lin & Kim (2016) [2]
	PE2: Participating in short video ads takes less time and effort	
	PE3: Short video ads can link products directly	
Ad Attitude	AT1: I like the sponsored advertisement section in short video	Taylor et al. (2011) [10]; Lin & Kim (2016) [2]
	AT2: I like pictures uploaded by ad sponsors on short videos	
	AT3: I’ll be positive about short video ads	
	AT4: I like the style of short video ads	
Purchase Intention	PI1: I will buy miscellaneous products shown on short video ads	Lin & Kim (2016) [2]
	PI2: I intend to purchase products shown on short video ads	
	PI3: I will recommend products shown on short videos to others	

**Table 4:** Statistics of construct items.

Variable	Item	Factor Loading	Cronbach’s α	CR	AVE	Mean	S. D.
Social Interaction	SI1	0.76	0.800	0.805	0.580	3.753	0.895
	SI2	0.70					
	SI3	0.82					
Intrusiveness	IR1	0.78	0.889	0.899	0.691	3.787	0.886
	IR2	0.84					
	IR3	0.89					
	IR4	0.81					
Informativeness	IN1	0.84	0.886	0.884	0. 717	2.354	0.974
	IN2	0.85					
	IN3	0.85					
Relevance	RE1	0.84	0.907	0.909	0.769	3.777	0.946
	RE2	0.90					
	RE3	0.89					
Perceived Usefulness	PU1	0.77	0.828	0.824	0.609	3.583	0.990
	PU2	0.76					
	PU3	0.81					
Perceived Ease of use	PR1	0.85	0.898	0.898	0.746	3.900	0.926
	PR2	0.90					
	PR3	0.84					
Ad Attitude	AT1	0.77	0.862	0.867	0.621	3.768	1.002
	AT2	0.81					
	AT3	0.80					
	AT4	0.77					
Purchase Intention	PI1	0.86	0.892	0.893	0.735	3.620	1.145
	PI2	0.90					
	PI3	0.81					

**Table 5:** Correlations between constructs (AVE and squared correlations).

	1	2	3	4	5	6	7	8
1 Social Interaction	0.762							
2 Intrusiveness	−0.122	0.831						
3 Informativeness	0.116	−0.237	0.847					
4 Relevance	0.329	−0.248	0.253	0.78				
5 Perceived Ease of Use	0.003	0.241	−0.23	0.163	0.864			
6 Perceived Usefulness	0.224	−0.114	0.101	0.293	−0.021	0.877		
7 Ad Attitude	0.194	−0.127	0.131	0.6	0.174	0.171	0.788	
8 Purchase Intention	0.113	−0.08	0.082	0.344	0.073	0.1	0.353	0.857

Note: The numbers in the diagonal row are square roots of the average variance extracted.

**Table 6:** Mediating effects analysis.

Path	Effect	Bootstrapping (2000 Bootstrap Samples)				Result
		Bias-Corrected		Percentile		
		95% CI		95% CI		
		Lower	Upper	Lower	Upper	
Social Interaction→PU→AT	total	0.104	0.418	0.095	0.407	Partial mediation
	direct	0.024	0.348	0.014	0.33	
	indirect	0.014	0.166	0.013	0.162	
Intrusiveness→PU→AT	total	−0.355	−0.088	−0.355	−0.087	Partial mediation
	direct	−0.285	−0.02	−0.282	−0.018	
	indirect	−0.134	−0.018	−0.133	−0.017	
Informativeness→PU→AT	total	0.118	0.367	0.113	0.358	Partial mediation
	direct	0.025	0.308	0.018	0.299	
	indirect	0.011	0.164	0.01	0.162	
Relevance→PU→AT	total	0.079	0.366	0.071	0.36	Failed
	direct	0.017	0.32	0.008	0.313	
	indirect	−0.003	0.159	−0.01	0.147	
Perceived Ease of Use →PU→AT	total	0.129	0.467	0.123	0.459	Partial mediation
	direct	0.051	0.378	0.035	0.363	
	indirect	0.024	0.188	0.02	0.184	
Social Interaction→PU→PI	total	−0.081	0.287	−0.082	0.287	Failed
	direct	−0.164	0.21	−0.163	0.21	
	indirect	0.015	0.199	0.009	0.176	
Intrusiveness→PU→PI	total	−0.272	0.072	−0.273	0.068	Failed
	direct	−0.22	0.133	−0.221	0.133	
	indirect	−0.144	−0.012	−0.14	−0.011	
Informativeness→PU→PI	total	−0.138	0.189	−0.126	0.197	Failed
	direct	−0.2	0.135	−0.193	0.143	
	indirect	0.01	0.161	0.006	0.155	
Relevance→PU→PI	total	0.013	0.353	0.013	0.354	Failed
	direct	−0.059	0.3	−0.05	0.308	
	indirect	−0.001	0.161	−0.01	0.141	
Perceived Ease of Use→PU→PI	total	0.099	0.479	0.085	0.466	Partial mediation
	direct	0.009	0.383	0.002	0.378	
	indirect	0.018	0.195	0.014	0.185	
Perceived Usefulness→AT→PI	total	0.216	0.615	0.218	0.618	Full mediation
	direct	−0.007	0.511	−0.015	0.507	
	indirect	0.026	0.368	0.024	0.358	

**Table 7:** Summary of hypotheses validation.

Number	Hypothesis	Validation Result
H1	Social interaction of health-related short video ads positively affects users’ perceived usefulness (PU) of ads.	Established
H2	Intrusiveness of health-related short video ads negatively affects users’ perceived usefulness (PU) of ads.	Established
H3	Informativeness of health-related short video ads positively affects users’ perceived usefulness (PU) of ads.	Established
H4	Relevance of health-related short video ads positively affects users’ perceived usefulness (PU) of ads.	Established
H5	Users’ perceived usefulness of health-related short video ads positively affects their attitudes toward ads.	Established
H6	Users’ perceived usefulness of health-related short video ads positively affects their purchase intentions.	Established
H7	Users’ perceived ease of use of health-related short video ads positively affects their perceived usefulness.	Established
H8	Users’ perceived ease of use of health-related short video ads positively affects their attitudes toward ads.	Not established
H9	Users’ attitudes of health-related short video ads positively affect their purchase intentions.	Established

## References

[1] Alalwan A. (2018). Investigating the impact of social media advertising features on customer purchase intention. Int. J. Inf. Manag..

[2] Lin C., Kim T. (2016). Predicting user response to sponsored advertising on social media via the technology acceptance model. Comput. Hum. Behav..

[3] China Internet Network Information Center (CNNIC) The forty-first China statistical report on the development of the Internet. www.cnnic.net.cn/hlwfzyj/hlwxzbg/hlwtjbg/201801/P020180131509544165973.pdf.

[4] Wang X., Tian Y., Lan R., Yang W., Zhang X. (2019). Beyond the Watching: Understanding Viewer Interactions in Crowdsourced Live Video Broadcasting Services. IEEE Trans. Circuits Syst. Video Technol..

[5] Hamouda M. (2018). Understanding social media advertising effect on consumers’ responses: An empirical investigation of tourism advertising on Facebook. J. Enterprise Inf. Management..

[6] Krämer J., Schnurr D., Wohlfarth M. (2019). Winners, losers, and Facebook: The role of social logins in the online advertising ecosystem. Manag. Sci..

[7] Zhang H., Cao X., Ho J., Chow T. (2017). Object-level video advertising: An optimization framework. IEEE Trans. Ind. Inform..

[8] Ducoffe R. (1996). Advertising value and advertising on the web. J. Advert. Res..

[9] Khalis A., Mikami A. (2018). Talking face-to-Facebook: Associations between online social interactions and offline relationships. Comput. Hum. Behav..

[10] Taylor D., Lewin J., Strutton D. (2011). Friends, fans, and followers: Do ads work on social networks? How gender and age shape receptivity. J. Advert. Res..

[11] Sun X., Han M., Feng J. (2019). Helpfulness of online reviews: Examining review informativeness and classification thresholds by search products and experience products. Decis. Support Syst..

[12] Dehghani M., Niaki M.K., Ramezani I., Sali R. (2016). Evaluating the influence of YouTube advertising for attraction of young customers. Comput. Hum. Behav..

[13] Lee J., Hong I.B. (2016). Predicting positive user responses to social media advertising: The roles of emotional appeal, informativeness, and creativity. Int. J. Inf. Manag..

[14] Stafford M., Faber R. (2015). Advertising, Promotion, and New Media.

[15] Pintado T., Sanchez J., Carcelén S., Alameda D. (2017). The effects of digital media advertising content on message acceptance or rejection: Brand trust as a moderating factor. J. Internet Commer..

[16] Jung A. (2017). The influence of perceived ad relevance on social media advertising: An empirical examination of a mediating role of privacy concern. Comput. Hum. Behav..

[17] Panniello U., Hill S., Gorgoglione M. (2016). The impact of profit incentives on the relevance of online recommendations. Electron. Commer. Res. Appl..

[18] Kahneman D. (1973). Attention and Effort. Prentice-Hall Series in Experimental Psychology.

[19] Fan S., Lu Y. (2017). Sumeetgupta, Social media in-feed advertising: The impacts of consistency and sociability on ad avoidance. Proc. PACIS.

[20] Mao E., Zhang J. (2017). What affects users to click on display ads on social media? The roles of message values, involvement, and security. J. Inf. Priv. Secur..

[21] Zeng F., Huang L., Dou W. (2009). Social factors in user perceptions and responses to advertising in online social networking communities. J. Interact. Advert..

[22] Windels K., Heo J., Jeong Y., Porter I., Jung A., Wang R. (2018). My friend likes this brand: Do ads with social context attract more attention on social networking sites?. Comput. Hum. Behav..

[23] Davis F. (1989). Perceived usefulness, perceived ease of use and user acceptance of information technology. Mis. Q..

[24] Zhao J., Zhu C., Peng Z., Xu X., Liu Y. (2018). User willingness toward knowledge sharing in social networks. Sustainability.

[25] Marakarkandy B., Yajnik N., Dasgupta C. (2017). Enabling internet banking adoption: An empirical examination with an augmented technology acceptance model (TAM). J. Enterp. Inf. Manag..

[26] Zhao J., Fang S., Jin P. (2018). Modeling and quantifying user acceptance of personalized business modes based on TAM, trust and attitude. Sustainability.

[27] Demangeot C., Broderick A.J. (2010). Consumer perceptions of online shopping environments: A gestalt approach. Psychol. Mark..

[28] Venkatesh V., Morris M., Davis G., Davis F. (2003). User acceptance of information technology: Toward a unified view. Mis. Q..

[29] Venkatesh V., Thong J., Xu X. (2012). Consumer acceptance and use of information: Extending the unified theory of acceptance and use of technology. Mis. Q..

[30] Kim S., Lee K., Hwang H., Yoo S. (2016). Analysis of the factors influencing healthcare professionals’ adoption of mobile electronic medical record (EMR) using the unified theory of acceptance and use of technology (UTAUT) in a tertiary hospital. BMC Med Inform. Decis. Mak..

[31] Im I., Hong S. (2011). Kang, An international comparison of technology adoption: Testing the UTAUT model. Inf. Manag..

[32] Dwivedi Y., Rana N., Jeyaraj A., Clement M., Williams M. (2019). Re-examining the unified theory of acceptance and use of technology (UTAUT): Towards a revised theoretical model. Inf. Syst. Front..

[33] Kreijns K., Kirschner P.A., Jochems W., van Buuren H. (2007). Measuring perceived sociability of computer-supported collaborative learning environments. Comput. Educ..

[34] McCoy S., Everard A., Galletta D., Moody G. (2017). Here we go again! The impact of website ad repetition on recall, intrusiveness, attitudes, and site revisit intentions. Inf. Manag..

[35] Hühn A., Khan V., Ketelaar P., Jonathan V., Konig R., Rozendaal E., Batalas N., Markopoulos P. (2017). Does location congruence matter? A field study on the effects of location-based advertising on perceived ad intrusiveness, relevance & value. Comput. Hum. Behav..

[36] Du B., Wang Z., Zhang L., Zhang L., Liu W., Shen J., Tao D. (2017). Exploring representativeness and informativeness for active learning. IEEE Trans. Cybern..

[37] de Mooij M., Hofstede G. (2010). The Hofstede model: Applications to global branding and advertising strategy and research. Int. J. Advert..

[38] Nie H., Yang Y., Zeng D. (2019). Keyword generation for sponsored search advertising: Balancing coverage and relevance. IEEE Intell. Syst..

[39] Can L., Kaya N. (2016). Social networking sites addiction and the effect of attitude towards social network advertising. Procedia-Soc. Behav. Sci..

[40] Tran T. (2017). Personalized ads on Facebook: An effective marketing tool for online marketers. J. Retail. Consum. Serv..

[41] Animesh A., Pinsonneault A., Yang S., Oh W. (2011). An odyssey into virtual worlds: Exploring the impacts of technological and spatial environments on intention to purchase virtual products. Mis. Q..

[42] Chowdhury H., Parvin N., Weitenberner C., Becker M. (2006). Consumer attitude toward mobile advertising in an emerging market: An empirical study. Int. J. Mob. Mark..

[43] Wang N., Shen X., Sun Y. (2013). Transition of electronic word-of-mouth services from web to mobile context: A trust transfer perspective. Decis. Support Syst..

[44] Podsakoff P., MacKenzie S., Lee J., Podsakoff N. (2003). Common method biases in behavioral research: A critical review of the literature and recommended remedies. J. Appl. Psychol..

[45] Nunnally J., Bernstein I. Psychometric Theory (3rd Ed.). McGrawHill, New York, USA, 1994.

[46] Hair J., Black C., Babin J., Anderson R., Tatham R. (1998). Multivariate Data Analysis.

[47] Fornell C., Larcker D. (1981). Evaluating structural equation models with unobservable variables and measurement error. J. Mark. Res..

[48] Preacher K., Hayes A. (2008). Asymptotic and resampling strategies for assessing and comparing indirect effects in multiple mediator models. Behav. Res. Methods.

[49] Amazon Mechanical Turk. https://www.mturk.com.

[50] Zhao J., Wang J., Fang S., Jin P. (2018). Towards sustainable development of online communities in the big data era: A study of the causes and possible consequence of voting on user reviews. Sustainability.

